# Cirrhosis of liver: Interference of serpins in quantification of SERPINA4 – A preliminary study

**DOI:** 10.1016/j.plabm.2017.10.002

**Published:** 2017-10-07

**Authors:** Krishna Sumanth Nallagangula, K.N. Shashidhar, V. Lakshmaiah, C. Muninarayana

**Affiliations:** Department of Biochemistry, Sri Devaraj Urs Medical College, SDUAHER, Tamaka, Kolar, Karnataka, India

## Abstract

**Background:**

Cirrhosis of liver is a pathological condition, wherein functions of liver are impaired by chronic liver exploitations. Due to decrease in synthetic capacity, expressions of plasma proteins tend to decrease in blood stream. Serpins (Serine protease inhibitors) are class of plasma proteins expressed from liver with structural similarities and diverse functions. SERPINA4 (Kallistatin) is a multifunctional serpin clade A protein expressed from liver and concentration in serum is the reflection of extent of liver dysfunction.

**Objective:**

To identify interference of other serpins by immunological cross reactivity with SERPINA4 in cirrhotic liver and healthy subjects.

**Materials and methods:**

Blood samples were collected from 20 subjects (10 cirrhotic liver, 10 healthy) from R.L. Jalappa Hospital and Research Centre, Kolar, Karnataka, India. Separation of proteins was carried out by SDS-PAGE. Cross reactivity study was analyzed using western blot.

**Results:**

Proteins present in cirrhotic liver and healthy subject's serum were separated by SDS PAGE. There was no band detection on both (cirrhotic liver and healthy) PVDF (polyvinylidene diflouride) membranes. However, a significant band was observed with recombinant kallistatin.

**Conclusion:**

Structurally similar serpins with minor amino acid sequence similarities did not show any immunological cross reactivity with SERPINA4 due to non identical epitope in cirrhotic liver and healthy subjects. Present study revealed that there is no interference of serpins for immunological reactions in quantitative estimation of kallistatin which needs further validation.

## Highlights

• To rule out structurally similar Serpins interference in SERPINA4 quantitative estimations by immunological interactions.
• A preliminary study for the evaluation of method in evolution of biomarker Kallistatin for Cirrhosis of Liver.
• Case control study using analytical methods SDS-PAGE and western blot.

## 1 Introduction

Cirrhosis of liver is a pathological condition characterized by diffuse fibrosis, severe disruption of intra hepatic arterial and venous flow, portal hypertension and finally liver failure resulting from varied etiologies of chronic liver diseases [1]. Despite different etiological factors, pathological characteristics, degeneration, necrosis of hepatocytes, replacement of parenchyma by fibrotic tissue, regenerative nodules; loss of liver functions are common [2]. Liver is a major organ with synthetic capacity to produce plasma proteins. Reduction in concentration of plasma proteins is reflected as decreased hepatic synthesis [3].

Serpins (Serine Protease Inhibitors) are class of plasma proteins that have similar structure and diverse functions. Serpins are divided into clades based on sequence similarities. In humans (clades A to I), 36 serpin coding genes and 5 pseudogenes are identified based on phylogenetic relationship [4]. Extracellular clade A molecules are localized on chromosomes 1, 14 and X. Intracellular clade B serpins are localized on chromosome 6 and 18 [5]. Serpins are interrelated due to highly conserved core structure [6]. Majority of clade A serpins are localized on chromosome 14 which are expressed from liver.

SERPINA1, (α1-antitrypsin) is an inhibitor of neutrophil elastase [5]. Pseudogene SERPINA2 indicates an ongoing process of pseudogenization [7]. Antichymotrypsin, SERPINA3 is an inhibitor of chymotrypsin and cathepsin G found in blood, liver, kidney and lungs [5]. SERPINA5 inhibits active C protein and are expressed from liver [4]. Non inhibitory hormone binding protein, SERPINA6 is a cortisol transporter [8]. SERPINA9 which is expressed from liver plays an important role in maintaining native B cell [4]. The inhibitory protein of activated coagulation factors Z and XI is SERPINA10 [5]. SERPINA11 is a pseudogene and uncharacterized [4]. SERPINA12 is an inhibitory protein of kallikrein and plays a role in insulin sensitivity [9].

Kallistatin (SERPINA4, serpin family A member 4, tissue kallikrein inhibitor), belongs to clade A serpins encoded by the SERPINA4 gene with 5 exons and 4 introns mapped to chromosome 14q31-32.1 in humans and expressed from liver cell lines (Hep G2 and Hep 3B). It is an acidic glycoprotein with a molecular weight of 58kD and isoelectric pH ranges from 4.6 to 5.2 [10], [11]. Apart from inhibitory action on human tissue kallikrein, it is a potent vasodilatory protein [12]. Kallistatin is involved in prevention of cancer, cardiovascular disease and arthritis through the effects of antiangiogenic, anti-inflammatory, antiapoptotic and antioxidative properties [13].

Kallistatin concentration in serum depends on the degree of severity of different chronic liver diseases (fibrosis, cirrhosis and hepatocellular carcinoma) [13]. Interference of other serpins with antibodies may give a significant false positive/negative value in quantitative estimations of kallistatin, which may mislead in assessment of extent of the disease. Hence, in the present study, an attempt has been made to identify immunological cross reactivity between kallistatin and other serpins in cirrhotic liver and compared with healthy subjects.

## 2 Materials and methods

### 2.1 Samples

Blood samples were collected from 20 subjects: 10 clinically and diagnostically proven cirrhotic liver subjects with varying degree, age and gender matched 10 healthy subjects **(**Table 1**)** from R. L. Jalappa Hospital and Research Centre, Kolar, Karnataka, India. Collection of blood samples from cirrhotic liver and healthy subjects was carried out after obtaining informed consent and study is approved by Institutional Ethical Committee (DMC/KLR/IEC/61/2016-17).

**Table 1:** Details of 20 blood samples (10 liver cirrhotic subjects, age and gender matched 10 healthy subjects) used for SDS PAGE and Western blot.

**Sample ID**	**Gender**	**Age**	**Etiology**	**Sample ID**	**Gender**	**Age**	**Etiology**
C1	M	36	NA	D1	M	36	ALD
C2	M	28	NA	D2	M	28	ALD
C3	M	62	NA	D3	M	62	ALD
C4	F	26	NA	D4	F	26	NAFLD
C5	M	35	NA	D5	M	35	ALD
C6	F	26	NA	D6	F	26	NAFLD
C7	M	70	NA	D7	M	70	ALD
C8	M	30	NA	D8	M	30	ALD
C9	M	62	NA	D9	M	62	ALD
C10	M	30	NA	D10	M	30	ALD

Abbreviations: C: Control; D: Diseased (Cirrhosis of liver); M: Male; F: Female; NA: Not Applicable; ALD: Alcoholic Liver Disease; NAFLD: Non Alcoholic Fatty Liver Disease.

### 2.2 Serum preparation

Serum was collected from clotted blood using serum separator tubes centrifuged at 4000 rpm for 10 min. Serum was stored at − 20 °C for further analysis. All the samples were used to find cross reactivity of other serpins with kallistatin by western blot after protein segregation by sodium dodecyl sulphate polyacrylamide gel electrophoresis (SDS-PAGE).

### 2.3 Reagents

Primary monoclonal antibodies specific for kallistatin along with secondary antibodies and recombinant kallistatin were procured from R&D systems, USA. Other chemicals of analytical grade were procured from Bio-Rad and Sigma Aldrich, USA.

### 2.4 SDS-PAGE analysis

SDS gels were prepared as per standard protocol. Cirrhotic liver and healthy subject's serum samples were loaded in different gels and SDS-PAGE was carried with duplication at 25 mA (2 gels run @ 50 mA) in 1X SDS running buffer. After electrophoresis, gels were incubated in fixing solution (7% acetic acid, 10% methanol) at room temperature for 20 min. At this point, the gels were transferred onto a PVDF (polyvinylidene diflouride) membrane for western blot and duplicate gels were subjected for staining with colloidal Coomassie brilliant blue in a shaker at room temperature for 2 h. Excess staining solution was removed and the gels were washed with 10% acetic acid and placed in deionized water for destaining till the appearance of bands [14], [15].

### 2.5 Western blot analysis

Proteins separated by SDS-PAGE were transferred onto PVDF membranes using a Transblot-Blot SD semi dry transfer cell (Bio-Rad) at 15 V for 2 h (1X transfer buffer: Tris/Glycine with 20% Methanol). After transfer, PVDF membranes were kept for blocking using blocking buffer (5% skimmed milk powder in 1X PBST) and incubated over night at 4 °C. After overnight blocking, PVDF membranes were washed with 1X PBST thrice for 3 min each. Primary antibodies were diluted (1:100) and PVDF membranes were incubated in diluted primary antibody solution at room temperature with slow shaking on rocker for 2–3 h. PVDF membranes were washed with 1X PBST thrice for 3 min each.

Secondary antibody was diluted (1:5000) and PVDF membranes were incubated in diluted secondary antibody solution at room temperature with slow shaking on rocker for 2–3 h. After incubation, PVDF membranes were washed with 1X PBST thrice for 3 min each. 12.5 mL Tris buffer (pH 7.35), 30 µl of 30% H_2_O_2_, a pinch of DAB were added into detection tray, mixed well and PVDF membranes were kept into the tray. The tray was gently shaken for a period of 10 min until the colour developed in the control lane [16], [17]. SDS-PAGE and western blot were repeated with pooled and concentrated cirrhotic liver and healthy serum samples along with recombinant kallistatin.

### 2.6 Concentration of serum proteins by dialysis using solid sucrose

Dialyzing tube containing serum to be concentrated is coiled up in a beaker and covered with commercial sucrose for 4 h. The liquid accumulated outside the dialyzing bag was poured off. Tubing was removed from the sugar at the end of 4 h and is tied off above the solution placed in water to dialyze away the sugar [18].

## 3 Results

### 3.1 Separation of proteins

Since SDS-PAGE is an efficient tool for separation of proteins based on molecular weight, proteins in serum were separated in both diseased (D1–D10) and healthy (C1–C10) gels along with corresponding molecular weight marker. Recombinant kallistatin was spotted on another SDS-PAGE with pooled and concentrated samples of cirrhotic liver and healthy subjects.

### 3.2 Immunological cross reactivity analysis

**Fig. 1:**
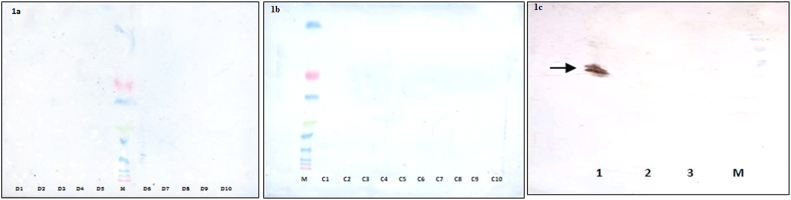
**a:** Western blot with diseased serum; M: Marker; D: Diseased subjects (Cirrhosis of Liver); **b:** Western blot with control serum; M: Marker; C: Healthy subjects; **c:** Western blot with Recombinant kallistatin; 1: Recombinant kallistatin; 2: Pooled and concentrated diseased serum; 3: Pooled and concentrated control serum; 4: Marker; **Arrow: Detection of band with recombinant kallistatin**.

Western blot analysis allowed identification of cross reactivity of serpins in diseased and healthy samples using monoclonal antibodies specific for kallistatin followed by secondary antibodies conjugated with HRP. No bands were observed on PVDF membranes of diseased **(**Fig. 1**a)** as well as healthy **(**Fig. 1**b)** samples. However, a significant band was observed with recombinant kallistatin. There was no band detection with pooled and concentrated samples of diseased and healthy **(**Fig. 1**c)** indicating that there is no cross reactivity of other serpins with kallistatin.

## 4 Discussion

Serpins are broadly distributed family of protease inhibitors which circulates in blood and are mainly expressed from liver [19]. Highly conserved similar structure (native, monomeric, active, latent, cleaved, delta and polymeric proteins) of serpins are crucial for their inhibitory function and play an important role in haemostasis and fibrinolysis [4], [6]. These proteins are suicide or single use inhibitors that use conformational changes to inhibit target enzymes [20]. Inhibitor binds tightly to a protease by incorporating reactive centre loop of inhibitor into β sheet of the enzyme by forming SDS and heat stable complex [6].

A highly conserved secondary and tertiary structure is the main criteria for the classification with modest amino acid similarities [4]. Despite chromosomal proximity, these genes have divergent function [21]. Serpin genes are present in clusters on same chromosome with common precursor. The human genes encoding α1-antitrypsin, corticosteroid-binding globulin, α1-antichymotrypsin and protein C inhibitor are mapped to the chromosome 14q32.1. Kallistatin is also mapped within the region on the same chromosome [22], [23].

In spite of similarity in chemical properties having minor amino acids sequence resemblance and mapped on same gene, our study did not show any cross reactivity between serpin class proteins in cirrhotic liver and healthy subjects which may be attributed due to absence of identical epitope among serpins. Cross reactivity occurs when two different serpins share an identical epitope. Epitope comprises approximately 15 amino acids of which 5 amino acids influence strongly for binding to definite paratope of Fab region on variable domain of antibody [24]. Due to the absence of identical epitope among serpins might be reason for no cross reactivity in cirrhotic liver and healthy subjects. There will be reduced expression of serpin proteins into blood stream due to decreased synthetic function of liver in cirrhotic liver subjects.

Molecular basis of polymerization is induced by mutations or mild denaturation which is common for all serpins. The conformational change in the serpin structure is crucial for functions and which also is susceptible reason for mutations [6]. Mutations which bring about polymerization can also occur anywhere in the serpin and leads to formation and accumulation of stable polymers with similar properties [25], [26]. Serpin polymerization can also occur through domain swapping as recorded in antithrombin, α-1 antitrypsin and neuroserpin, which needs further studies to evaluate domain swapping polymerization of entire serpin family proteins, [27], [28], [29]. Polymerization leads to reduction in serpin secretion with qualitative changes in protein structure [6]. The etiological factors of cirrhosis of liver may not induce polymerization which directs to share identical epitope of serpin family proteins. This may be the reason why no cross reactivity was observed in cirrhotic liver subjects in our study.

Even though, incidence of diseases caused by serpin polymerization is rare, homozygous mutations in SERPINA1 gene (α1 antitrypsin) is associated with liver disease including cirrhosis. Human variants of serpin genes has been found in large number as a resultant of mutations which are associated with many diseases **(**Table 2**)** [4], [30]. SERPINA1 alone has 1411 SNPs; SNPs for SERPINA4 are 906 in NCBI's dbSNP database (Accessed: July 2017). Mutational studies in terms of cross reactivity, for identification of identical epitope, might be difficult at this point because of huge diversity of serpins.

**Table 2:** Classification of serpin clade A, chromosomal location, polymerization associated diseases.

**Sl. no.**	**Name**	**Symbols**	**Synonyms**	**Chromosome**	**Associated diseases**
1	SERPINA1	PI	α-1-antitrypsin, AAT	14q32.13	Emphyesma, Chronic Liver Disease, Vasculitis
2	SERPINA2	PIL	ATR, ARGS	14q32.13	–
3	SERPINA3	AACT	ACT	14q32.13	Emphyesma
4	SERPINA4	PI4	KST, KAL, KLST, Kallistatin	14q32.13	Renal and Cardiovasular Injury
5	SERPINA5	PLANH3	PA13, PROCI	14q32.13	Angiodema, Papillary thyroid cancer
6	SERPINA6	CBG	–	14q32.13	Chronic fatigue syndrome
7	SERPINA7	TBG	–	Xq22.3	Deficiency results in hypothyroidism
8	SERPINA8	AGT	–	1q42.2	Certain varients linked to essential hypertension
9	SERPINA9	–	CENTERIN	14q32.13	–
10	SERPINA10	–	PZI	14q32.13	Risk of venous thromboembolism, Pregnancy Complications
11	SERPINA11	–	–	14q32.13	–
12	SERPINA12	–	Vaspin, OL-64	14q32.13	Associated with Insulin resistance

Concentration of kallistatin is less in cirrhotic liver as well as in healthy subjects. Hence, the sensitivity of monoclonal antibodies (5 ng/lane, by manufacturer's instructions) might not detect kallistatin. In case of any cross reactivity, these antibodies may detect other serpins whose concentrations are in nanograms in serum. Use of more sensitive antibodies might detect kallistatin in cirrhotic liver as well as in healthy subjects and enhance successful immunological interactions of other serpins. For separation of proteins, 2 dimensional electrophoresis (2-DE) might be better option than single dimensional SDS-PAGE.

## 5 Conclusion

In the present study, no immunological cross reactivity was observed between serpins and SERPINA4 (kallistatin) due to the absence of identical epitope in cirrhotic liver and healthy subjects. Because of enormous diversity of serpins, validation of quantitative ELISA should be carried out to check interference of other factors (buffer components, sample matrix, compliment and rheumatoid factor) along with cross reactivity by using different types of antibodies. Further quantitative studies of Kallistatin may provide insights into potential diagnostic options for chronic liver diseases.
